# Malondialdehyde-Induced Post-Translational Modification of Human Hemoglobin

**DOI:** 10.1021/acs.jproteome.2c00764

**Published:** 2023-04-04

**Authors:** Dimitrios Tsikas

**Affiliations:** † Core Unit Proteomics, Institute of Toxicology, Hannover Medical School, Carl-Neuberg-Straße 1, 30625 Hannover, Germany

## Abstract

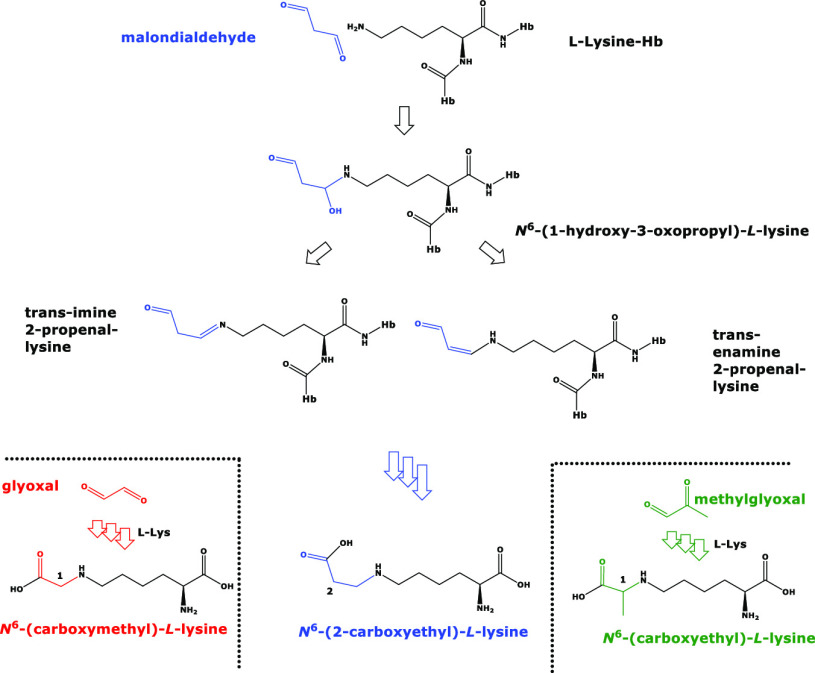

Lysine residues in proteins undergo multiple enzymatic and nonenzymatic post-translational modifications (PTMs). The terminal ε amine group of lysine residues in proteins is carbonylated chemically by carbonyl species such as glyoxal (GO; OCH–CHO, C_2_H_2_O_2_; MW 58) and methylglyoxal (MGO; OCH-C(=O)–CH_3_, C_3_H_4_O_2_; MW 72) that are derived from the metabolism of endogenous substances including glucose. The dicarbonyl species malondialdehyde (MDA, OCH–CH_2_–CHO, C_3_H_4_O_2_; MW 72) is generated by enzymatic and nonenzymatic peroxidation of polyunsaturated fatty acids (PUFAs). GO, MGO, and MDA occur in biological systems in their free forms and in their conjugated forms adducted to free amino acids and amino acid residues in proteins, notably to lysine. MDA is a C–H-acidic acid (p*K*_a_, 4.45). Biological MDA is widely used as a biomarker of lipid peroxidation. The most frequently analyzed biological samples for MDA are plasma and serum. Reportedly, MDA concentrations in plasma and serum samples of healthy and ill humans range by several orders of magnitude. The most severe preanalytical contributor is artificial formation of MDA in lipid-rich samples such as plasma and serum. In very few publications, plasma MDA concentrations were reported to lie in the lower mM-range.

Amino acid residues in proteins undergo multiple post-translational modifications (PTMs).^1^ Biological malondialdehyde (MDA, OCH–CH_2_–CHO, C_3_H_4_O_2_; MW 72; p*K*_a_, 4.45^2^) is widely used as a biomarker of lipid peroxidation.^3,4^ Gönenç and colleagues reported average MDA plasma concentrations of 6.3 μM in breast cancer patients, of 5.9 μM in lung cancer patients, and of 2.3 to 2.7 μM in healthy controls,^5^ indicating elevated oxidative stress in cancer. In plasma of healthy Taiwanese college students, Hong et al.^6^ reported mean MDA plasma concentrations ranging between 0.4 μM and 2.1 μM as determined by four different methods. In contrast, Akbulut and colleagues reported average MDA plasma concentrations of 2.7 mM (reported in units of μmol/mL) in patients with early breast cancer and 2.2 mM (reported in units of mmol/mL) in controls.^7^ These MDA concentrations are almost 3 orders of magnitude higher compared to those reported by Gönenç et al.^5^ and Hong et al.^6^ Furthermore, in patients with rheumatoid arthritis, average MDA serum concentrations of the order of 550 M were described (reported in units of mmol/mL).^8^ To the best of the author’s knowledge, there are no other articles reporting plasma or serum MDA concentrations of the order or 2 mM^7^ and 500 M.^8^ Such extraordinarily high MDA concentrations are simply impossible in biological samples. They are most likely typos or resulted from miscalculation and misreport of units.

Biological MDA is not the sole substance for which highly diverging concentrations have been reported. Originally reported concentrations for a series of other physiological substances such as the nitric oxide (NO) metabolites *S*-nitrosothiols,^9−11^ nitro-fatty acids,^12^ and nitrite and nitrate^13^ have not been confirmed by the authors. In contrast, they have been in part corrected later as discussed by us.^9−13^ The most likely reasons for originally reported high concentrations of endogenous substances are preanalytical and analytical shortcomings.

In a recent paper published in *J. Proteome Res*, Chen and colleagues investigated the reaction of MDA with hemoglobin (Hb) *in vitro* in a phosphate buffer (0.1 M, pH 8.0) by NanoLC-NSI/MS/MS.^14^ The Hb concentration used in the experiments was 100 μM, whereas synthetic MDA was used at concentrations of 0, 100, 250, and 500 μM,^14^ resulting in Hb-MDA molar ratios of 1:1 to 1:5. Chen et al. stated in their article^14^ that the MDA concentrations used in their investigations are physiological. Chen et al.^14^ explained the use of 0–500 μM MDA concentrations, because such a range has been reported in some papers.^5−7^ Yet, the authors did not refer to other articles, including reviews on MDA and references therein, which also reported on MDA reference values and intervals in health and disease.^3,4,15^ High MDA concentrations (0–1 mM) were also used by other authors in *in vitro* investigations with human serum albumin (HSA) and human Hb, which was discussed as a study limitation.^1,16^

Reported highly diverging concentrations of MDA and other endogenous substances do not reflect physiological or pathological variations of the analytes. Several orders of magnitude differing concentrations of endogenous substances in biological sample are most likely due to human error. Making compromises by accepting any published concentration as physiological or pathological is not acceptable but misguiding in scientific research. Based on our present knowledge of circulating MDA concentrations in health and disease, MDA concentrations of 100 μM, 250 μM, and 500 μM must be regarded as entirely nonphysiological. Translation of observations obtained by using such high MDA concentrations to physiology or pathology are expected to be limited. A limitation of the study by Chen et al.^14^ are the Hb-MDA molar ratios used, i.e., 1:1 to 1:5. Considering an erythrocytic Hb concentration of 8 mM, the MDA concentrations to be used in order to achieve Hb-MDA molar ratios of 1:1 to 1:5 would be 8 mM to 40 mM^14^ and consequently irrational.

From a mechanistic point of view, the observations by Chen et al.^14^ suggest that MDA may react with Lys residues of Hb to form adducts of the propenal-type (*N*^6^-(3-oxoprop-1-en-1-yl)-lysine) and dihydropyridine-type. The addition of C_3_H_2_O (54 Da) from MDA on lysine residues of Hb suggests formation of Schiff’s bases in the propenal-type from the dehydration of the expected first intermediate reaction product *N*^6^-(1-hydroxy-3-oxoprop-yl)-lysine.

*In vitro*, in a phosphate buffer (0.1 M, pH 7.4) and in the absence of any reducing and oxidizing chemicals and enzymes, we observed that the reaction of free Lys with glyoxal (OCH–CHO) or methylglyoxal (OCH–C(=O)–CH_3_) forms carboxymethyllysine (Lys-N^ε^H–CH_2_–COOH) and carboxyethyllysine (CEL, Lys-N^ε^H–C(−CH_3_)H–COOH) in very low yield, respectively (Figure 1).^17^ CML and CEL are two advanced glycation end-products (AGEs) and occur physiologically in human plasma and urine in the lower μM-range.^18^

**Figure 1:**
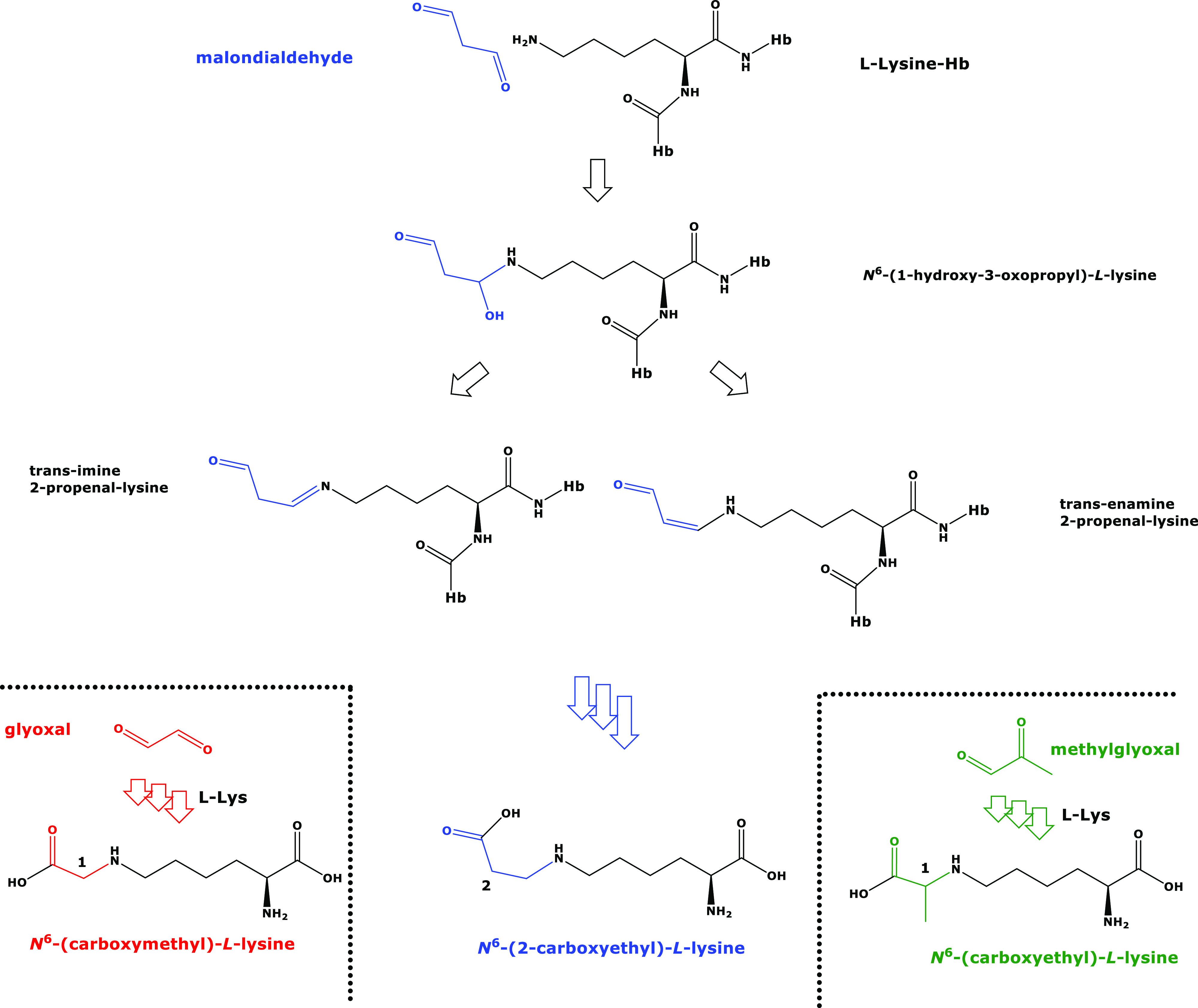
Proposed chemical structures and names of the reaction products of l-lysine with malondialdehyde, glyoxal, and methylglyoxal. The reaction products of the isomeric malondialdehyde (C_3_H_4_O_2_) and methylglyoxal (C_3_H_4_O_2_) with l-lysine are also isomeric. Hb, hemoglobin; l-Lys, l-lysine. Several arrows indicate multiple consecutive reactions. Color highlights free and incorporated malondialdehyde, glyoxal, and methylglyoxal.

Reduction of the CH=N-group of the Schiff’s bases and oxidation of their terminal aldehyde groups in MDA adducted to Lys residues of Hb, e.g., Hb-Lys-MDA, or of Lys-MDA released from proteolyzed Hb-Lys-MDA would form *N*^ε^-(1-carboxyyethyl)-l-lysine, CEL, Lys-N^ε^H–CH_2_CH_2_–COOH), an isomer of CEL (Figure 1). Should this occur *in vivo*, it would be very difficult to discriminate between CEL and CEL by chromatographic and mass spectrometric means. It is likely that CEL and CEL are measured in total and their origin, i.e., MDA and MGO would be undetermined.

Chen et al.^14^ found differences between smokers and nonsmokers. Noticeable is the almost 18-fold higher extent of formation between smokers and nonsmokers with respect to the MDA-modification on Lys-11 in the α-globin of Hb by the addition of C_3_H_2_O (54 Da) from MDA, i.e., α-^11^K^+54^, possibly indicating considerable site-specificity.

These observations need to be evaluated by using stable-isotope labeled Hb-MDA adducts as reported for several kinds of Hb adducts including epichlohydrin^19^ and MDA.^20^ In such investigations, potential differences in Hb concentrations between smokers and nonsmokers^21,22^ need to be considered and the Hb-MDA amounts need to be also corrected for the Hb concentrations measured in the subjects. Casado et al.^22^ found that the differences between smokers and nonsmokers with respect to Hb-MDA adducts were very moderate (1.1-fold). As to MDA itself, measurement in plasma, serum, or urine revealed contradictory smoking effects.^4^

## References

[ref1] Tsikas D. Post-translational modifications (PTM): analytical approaches, signaling, physiology and pathophysiology—Part II. Amino Acids 2022, 54 (4), 481–484. 10.1007/s00726-022-03164-2. 33929637 PMC8107173

[ref2] Osman M. M. The acidity of malondialdehyde and the stability of its complexes with nickel(II) and copper (II). Helv. Chim. Acta 1972, 55, 239–244. 10.1002/hlca.19720550127.

[ref3] Giustarini D.; Dalle-Donne D. D.; Tsikas D.; Rossi R. Oxidative stress and human diseases: Origin, link, measurement, mechanisms, and biomarkers. Crit. Rev. Clin. Lab. Sci. 2009, 46, 241–281. 10.3109/10408360903142326. 19958214

[ref4] Tsikas D. Assessment of lipid peroxidation by measuring malondialdehyde (MDA) and relatives in biological samples: Analytical and biological challenges. Anal. Biochem. 2017, 524, 13–30. 10.1016/j.ab.2016.10.021. 27789233

[ref5] Gönenç A.; Ozkan Y.; Torun M.; Simşek B. Plasma malondialdehyde (MDA) levels in breast and lung cancer patients. J. Clin. Pharm. Ther. 2001, 26 (2), 141–1444. 10.1046/j.1365-2710.2001.00334.x. 11350537

[ref6] Hong Y. L.; Yeh S. L.; Chang C. Y.; Hu M. L. Total plasma malondialdehyde levels in 16 Taiwanese college students determined by various thiobarbituric acid tests and an improved high-performance liquid chromatography-based method. Clin. Biochem. 2000, 33 (8), 619–625. 10.1016/S0009-9120(00)00177-6. 11166008

[ref7] Akbulut H.; Akbulut K. G.; Icli F.; Büyükcelik A. Daily variations of plasma malondialdehyde levels in patients with early breast cancer. Cancer Detect. Prev. 2003, 27 (2), 122–126. 10.1016/S0361-090X(03)00029-1. 12670523

[ref8] Hashemi G.; Mirjalili M.; Basiri Z.; Tahamoli-Roudsari A.; Kheiripour N.; Shahdoust M.; Ranjbar A.; Mehrpooya M.; Ataei S. A Pilot Study to Evaluate the Effects of Oral N-Acetyl Cysteine on Inflammatory and Oxidative Stress Biomarkers in Rheumatoid Arthritis. Curr. Rheumatol. Rev. 2019, 15 (3), 246–253. 10.2174/1573403X14666180926100811. 30255760

[ref9] Tsikas D. S-nitrosoalbumin and other S-nitrosothiols in the blood: is their quantity of no relevance?. Circ. Res. 2004, 94 (12), e106 10.1161/res.94.12.e106. 15217921

[ref10] Tsikas D.; Rossi R. Cocoa intake and blood pressure. JAMA 2007, 298 (16), 1862–1863. 10.1001/jama.298.16.1862-b. 17954536

[ref11] Tsikas D.; Zoerner A. A.; Gutzki F. M.; Rossi R. On the mercapturic acid pathway of nitric oxide: is S-nitrosoglutathione present in the bile?. Hepatology 2010, 52 (5), 1858–9. 10.1002/hep.23926. 20890894

[ref12] Tsikas D.; Zoerner A. A.; Mitschke A.; Gutzki F. M. Nitro-fatty acids occur in human plasma in the picomolar range: a targeted nitro-lipidomics GC-MS/MS study. Lipids 2009, 44 (9), 855–865. 10.1007/s11745-009-3332-4. 19701657

[ref13] Tsikas D.; Mikuteit M. N-Acetyl-L-cysteine in human rheumatoid arthritis and its effects on nitric oxide (NO) and malondialdehyde (MDA): analytical and clinical considerations. Amino Acids 2022, 54 (9), 1251–1260. 10.1007/s00726-022-03185-x. 35829920 PMC9372125

[ref14] Chen H. C.; Chen C. Y.; Fang Y. H.; Hung K. W.; Wu D. C. Malondialdehyde-Induced Post-translational Modifications in Hemoglobin of Smokers by NanoLC-NSI/MS/MS Analysis. J. Proteome Res. 2022, 21, 2947 10.1021/acs.jproteome.2c00442. 36375001

[ref15] Mas-Bargues C.; Escrivá C.; Dromant M.; Borrás C.; Viña J. Lipid peroxidation as measured by chromatographic determination of malondialdehyde. Human plasma reference values in health and disease. Arch. Biochem. Biophys. 2021, 709, 108941 10.1016/j.abb.2021.108941. 34097903

[ref16] Estévez M.; Padilla P.; Carvalho L.; Martín L.; Carrapiso A.; Delgado J. Malondialdehyde interferes with the formation and detection of primary carbonyls in oxidized proteins. Redox Biol. 2019, 26, 101277 10.1016/j.redox.2019.101277. 31352127 PMC6669345

[ref17] Baskal S.; Tsikas D. Free L-Lysine and Its Methyl Ester React. with Glyoxal and Methylglyoxal in Phosphate Buffer (100 mM, pH 7.4) to Form *N*^ε^-Carboxymethyl-Lysine, *N*^ε^-Carboxyethyl-Lysine and *N*^ε^-Hydroxymethyl-Lysine. Int. J. Mol. Sci. 2022, 23 (7), 3446 10.3390/ijms23073446. 35408807 PMC8998464

[ref18] Baskal S.; Bollenbach A.; Mels C.; Kruger R.; Tsikas D. Development, validation of a GC-MS method for the simultaneous measurement of amino acids, their PTM metabolites and AGEs in human urine, and application to the bi-ethnic ASOS study with special emphasis to lysine. Amino Acids 2022, 54 (4), 615–641. 10.1007/s00726-021-03031-6. 34251524 PMC9117344

[ref19] Bader M.; Rosenberger W.; Gutzki F. M.; Tsikas D. Quantification of N-(3-chloro-2-hydroxypropyl)valine in human haemoglobin as a biomarker of epichlorohydrin exposure by gas chromatography-tandem mass spectrometry with stable-isotope dilution. J. Chromatogr. B Analyt. Technol. Biomed. Life Sci. 2009, 877 (13), 1402–1415. 10.1016/j.jchromb.2008.11.028. 19059011

[ref20] Cipierre C.; Haÿs S.; Maucort-Boulch D.; Steghens J. P.; Picaud J. C. Malondialdehyde adduct to hemoglobin: a new marker of oxidative stress suitable for full-term and preterm neonates. Oxid. Med. Cell Longev. 2013, 2013, 694014 10.1155/2013/694014. 23844277 PMC3697782

[ref21] Ahmed N. J.; Husen A. Z.; Khoshnaw N.; Getta H. A.; Hussein Z. S.; Yassin A. K.; Jalal S. D.; Mohammed R. N.; Alwan A. F. The Effects of Smoking on IgE, Oxidative Stress and Haemoglobin Concentration. Asian Pac. J. Cancer Prev. 2020, 21 (4), 1069–1072. 10.31557/APJCP.2020.21.4.1069. 32334472 PMC7445955

[ref22] Casado Á.; Castellanos A.; López-Fernández M. E.; Ruiz R.; López Imedio E.; Castillo C.; Fernández-Nieto A. M. Determination of oxidative and occupational stress in palliative care workers. Clin. Chem. Lab. Med. 2011, 49 (3), 471–477. 10.1515/CCLM.2011.061. 21143019

